# Eight-gene metabolic signature related with tumor-associated macrophages predicting overall survival for hepatocellular carcinoma

**DOI:** 10.1186/s12885-020-07734-z

**Published:** 2021-01-07

**Authors:** Junyu Huo, Liqun Wu, Yunjin Zang, Hongjing Dong, Xiaoqiang Liu, Fu He, Xiao Zhang

**Affiliations:** 1 Liver Disease Center, The Affiliated Hospital of Qingdao University, No. 59 Haier Road, Qingdao, 266003 China; 2 Qingdao University, No. 308 Ningxia Road, Qingdao, 266071 China; 3 Linyi Central Hospital, Linyi, 276400 China

**Keywords:** Hepatocellular carcinoma, Tumor-associated macrophages, Metabolic, Prognostic, Signature

## Abstract

**Background:**

In recent years, the relationship between tumor-associated macrophages (TAMs) and solid tumors has become a research hotspot. This study aims to explore the close relationship of TAMs with metabolic reprogramming genes in hepatocellular carcinoma (HCC) to provide new methods of treatment for HCC.

**Methods:**

The study selected 343 HCC patients with complete survival information (survival time > = 1 month) in the Cancer Genome Atlas (TCGA) as study subjects. Kaplan-Meier survival analysis assisted in determining the relationship between macrophage infiltration and overall survival (OS), and Pearson correlation tests were used to identify metabolic reprogramming genes (MRGs) associated with tumor macrophage abundance. Lasso regression algorithms were used on prognosis-related MRGs identified by Kaplan-Meier survival analysis and univariate Cox regression analysis to construct a risk score; another independent cohort (including 228 HCC patients) from the International Cancer Genome Consortium (ICGC) was used to verify prognostic signature externally.

**Results:**

A risk score composed of 8 metabolic genes could accurately predict the OS of a training cohort (TCGA) and a testing cohort (ICGC). The risk score could be widely used for people with different clinical characteristics, and it is a predictor that is independent of other clinical factors that affect prognosis. As expected, compared with the low-risk group, the high-risk group exhibited an obviously higher macrophage abundance, together with a positive correlation between the risk score and the expression levels of three commonly used immune checkpoints (PD1, PDL1, and CTLA4).

**Conclusion:**

Our study constructed and validated a novel eight-gene signature for predicting HCC patient OS, which may contribute to clinical treatment decisions.

## Background

Growing evidence shows that tumor progression and metastasis are closely related to the tumor microenvironment [1, 2]. Once the tumor microenvironment is formed, many immune cells, such as T cells, myelogenic inhibitory cells, macrophages, and others, form the tumor microenvironment through chemotaxis [3]. Among these immune cells, tumor-associated macrophages (TAMs) are macrophages derived from the infiltration of peripheral blood monocytes into solid tumor tissues [4], and they are the most numerous inflammatory cell group in tumor stroma, accounting for approximately 30–50% of the total inflammatory cells [5, 6]. TAMs have been reported to have a remarkable effect on tumor occurrence, growth, invasion and metastasis [7–9], and thus they are receiving growing attention.

To adapt to the decreased nutrients and oxygen available in the tumor microenvironment (TME) and to maintain the rapid proliferation and material synthesis of tumor cells, a series of changes to the metabolic processes of tumor cells occur that lead to the increase in related metabolites, such as lactate, nitrous oxide, reactive oxygen species, prostaglandin, and arachidonic acid, in the tumor microenvironment, thus creating an inflammatory microenvironment [10, 11]. These changes also affect the function of tumor associated macrophages (TAMs), including changes in cytokines and angiogenic factors, which may prompt tumor progression and metastasis [12].

Hepatocellular carcinoma (HCC) contributes to more than 90% of all cases of liver cancer, and HCC ranks 2nd in cancer-related deaths worldwide [13]. Metabolic reprogramming has been reported to have a significant effect on the prognosis of HCC [14, 15]. However, there is no definitive understanding of the relationship between TAMs and metabolic reprogramming in HCC.

To examine how the infiltration of six types of immune cells affected HCC prognosis, the Tumor Immune Estimation Resource (TIMER) database was adopted in this study for the collection of tumor immune cell infiltration data. The patients with higher macrophage infiltration levels had a poor overall survival rate. We also identified differentially expressed metabolism-related genes (DEMRGs) between high-level and low-level macrophage infiltration groups of HCC patients and used these genes to build a prognostic signature.

## Methods

### Data collection

**Fig. 1:**
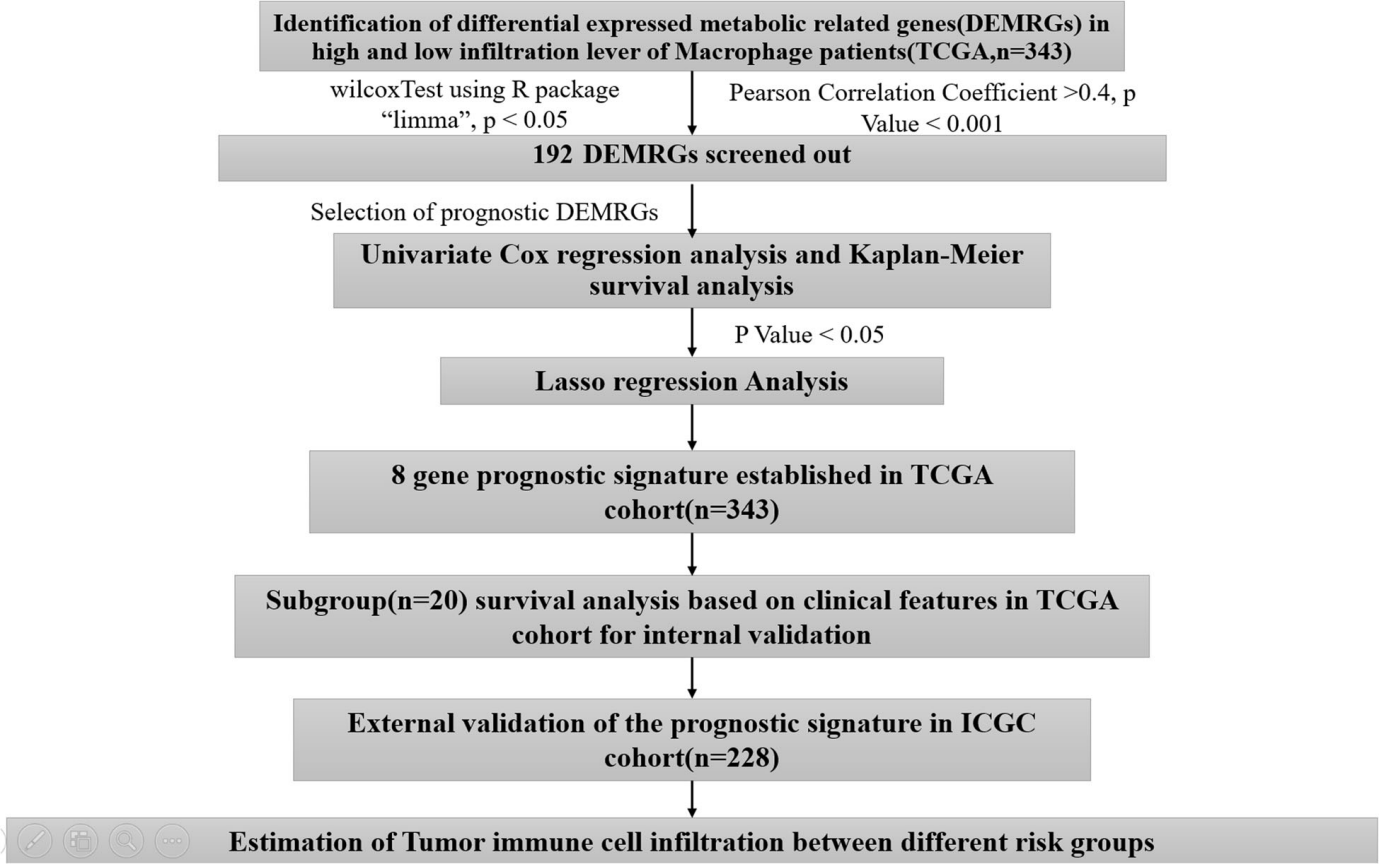
Workflow chart for this study

The immune infiltration data for tumors from the Cancer Genome Atlas (TCGA) were obtained from the TIMER (Tumor Immune Estimation Resource) website (http://timer.cistrome.org/) [16]. The RNA-sequencing data for the TCGA dataset, where 343 HCC cases were collected, were obtained from TCGA (https://portal.gdc.cancer.gov/); corresponding clinical data were acquired from the UCSC Xena website (https://xenabrowser.net/). The RNA-sequencing data including 228 HCC cases and their clinical information came from the International Cancer Genome Consortium (ICGC) (https://icgc.org/). With the purpose of eliminating the impact exerted by perioperative factors on patients’ survival, our study did not include patients who survived no more than 1 month. Sequencing data from TCGA and ICGC databases utilized in this study were collected from the same Illumina HiSeq_RNA-Seq platform, and the R package “combat” was used to remove batch effects. There is no need to obtain the approval of a local ethics committee since the above data can be accessed from public sources. This study was conducted following the regulations on the use of databases involved. Figure 1 displays the workflow.

### Identification of TAM-related metabolic genes (TRMGs)

According to the median value of immune cell infiltration, 343 HCC cases from the TCGA dataset were classified into two groups, which had high and low levels of infiltration. We extracted 2752 previously published genes related to metabolism that encode all the known human metabolic enzymes and transporters to perform the following analysis [17]. Then, we used the Wilcoxon test in the R package “limma” to identify metabolism-related genes (DEMRGs) that were expressed differently by the two groups. How gene expression correlated with estimated immune infiltration was examined by the Pearson correlation test. A significant correlation with TAM infiltration was identified by a *p*-value of smaller than 0.001 and a Pearson correlation coefficient of larger than 0.4. Kyoto Encyclopedia of Genes and Genomes (KEGG) pathway enrichment analysis of TRMGs was conducted by the R package “clusterprofiler”, where *P* < 0.05 was the threshold.

### Identification of prognostic TRMGs

The association of prognosis in the TCGA database with TRMGs was examined by Kaplan-Meier survival analysis and univariate Cox regression analysis. *P*-values < 0.05 were used as selection criteria for both methods.

### Construction of a risk score predict overall survival in the TCGA cohort

To construct a risk score, a lasso regression algorithm was performed on the prognosis-associated TRMGs identified by Kaplan-Meier survival analysis and univariate Cox regression analysis. It involved confirming the optimal penalty parameter λ related to the smallest 10-fold cross-validation in the TCGA dataset. In terms of the signature, the risk score was calculated as the sum of each mRNA coefficient * each mRNA expression. The formula assisted in computing the risk score specific to each patient in TCGA and ICGC datasets. With the median value as the unified cutoff, patients of the TCGA cohort were classified into low- and high-risk groups. The prediction accuracy of our risk signature for 0.5/1/3/5-year survival was assessed by the R package “survival ROC”, which generated the time-dependent receiver operating characteristic (ROC) curves. The Kaplan-Meier curves generated with the R package “suvminer” were used to compare patients’ OS in different groups using a log-rank test. The R package “glmnet” was utilized in this study to conduct lasso regression analysis.

### Internal validation of the prognostic signature in the TCGA cohort

Patients differing in clinical features (vascular invasion, body mass index, age, gender, race, histological grade, ajcc-tnm stage, individual tumor status, presence of new tumors after initial treatment, and previous malignancy history) are subject to Kaplan-Meier survival analysis to verify whether the prognostic model could be used to predict different populations’ prognoses.

### External validation of prognostic signature in ICGC cohort

The same formula mentioned above was used to calculate each patient’s risk score, resulting in high-risk and low-risk groups in the same way. Patients with different clinical features (previous malignancy, stage, age, gender) were also subject to Kaplan-Meier survival analysis.

### Independence validation of the prognostic signature

Based on univariate and multivariate Cox regression analyses, the risk score and clinicopathological features were obtained to determine whether the risk score could be used as an independent predictor of HCC prognosis.

### Correlation analysis between risk score and clinicopathology

We conducted chi-square tests on different risk groups for correlation analysis regarding clinical features. P less than 0.05 represented statistical significance.

### Estimation of immune infiltration

Two algorithms were used to estimate immune infiltration in different risk groups: TIMER, which is a method for estimating the abundance of 6 types of tumor-infiltrating immune cells (dendritic cells, macrophages, CD8 T cells, CD4 T, neutrophils, and B cells) [18]; and CIBERSORT-ABS, which is a methodology based on the gene expression profile for evaluating the absolute abundance of 22 immune cell populations [16].

### Gene set enrichment analysis

To clarify possible mechanisms underlying the prognostic signatures of patients with HCC, HCC patients in both TCGA and ICGC cohorts were subject to Gene Set Enrichment Analysis (GSEA). An annotated gene set file “c2.cp.kegg.v7.0.symbols.gmt” was used as reference. The threshold was confirmed as FDR < 0.25 and NOM *p*-value < 0.05.

### Statistical analysis

SPSS Statistics 25 (https://www.ibm.com/products/software) together with R v.3.6.1 (https://www.r-project.org/) was employed for all statistical analyses. Mann-Whitney U-tests and Unpaired Student’s t-tests assisted in comparing two groups containing variables with normal distributions and two groups containing variables with nonnormal distributions, respectively. To compare three groups, one-way analysis of variance served as the parametric method, and Kruskal-Wallis tests of variance were used as the nonparametric method. Fisher’s exact tests or chi-square tests assisted in analyzing the contingency table variables. Kaplan-Meier survival analysis was performed, together with log-rank tests for comparison. A univariate Cox proportional hazards regression model assisted in estimating the hazard ratios exhibited by univariate analyses. Statistical significance was identified by a two-tailed *P*-value < 0.05 [19].

## Results

### Identification and annotation of TRMGs

**Fig. 2:**
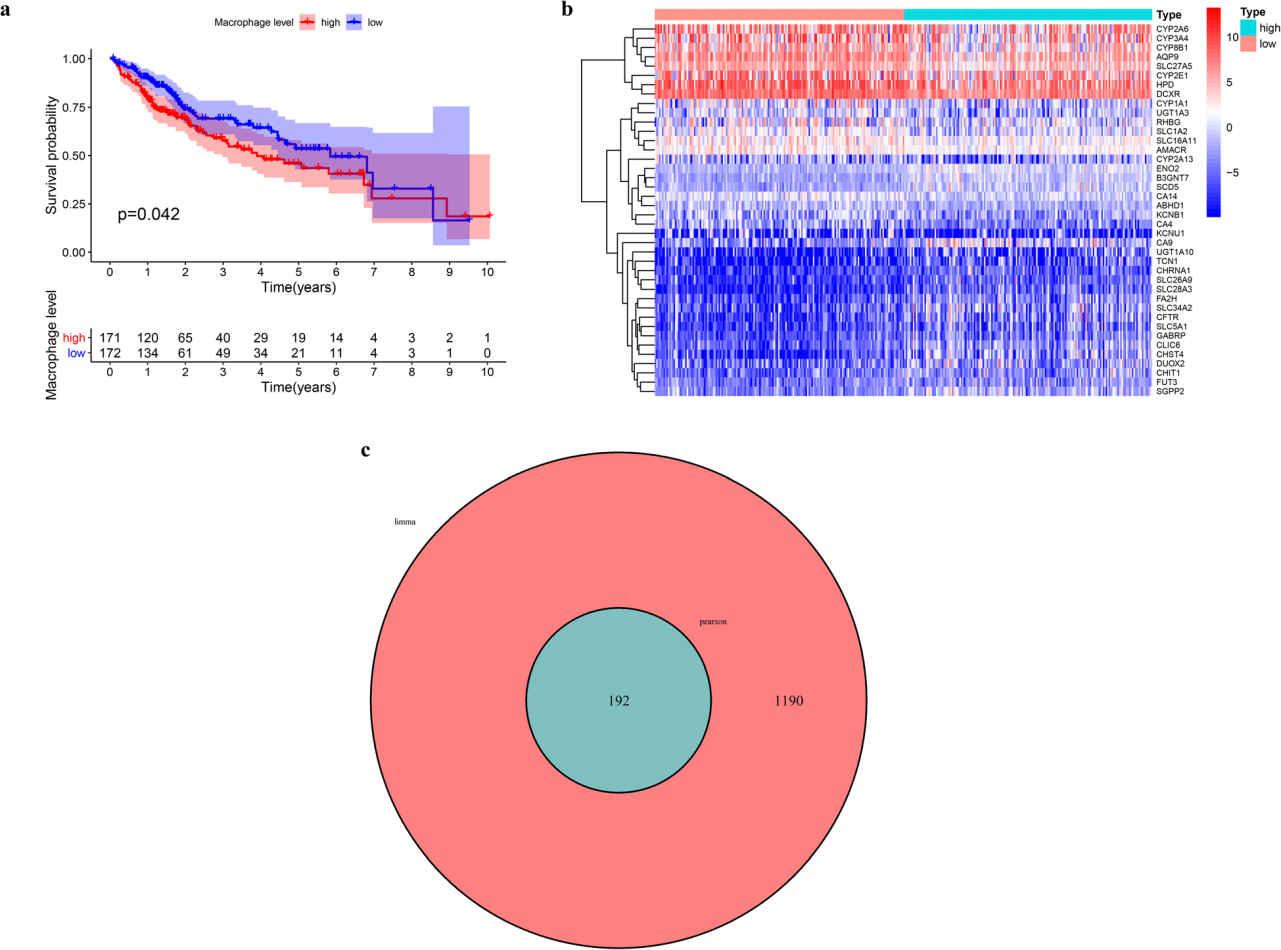
Identification of tumor-associated macrophage-related metabolic genes. **a** Kaplan-Meier survival curve showing the relationship between macrophage abundance and overall survival. **b** Heat map of differentially expressed metabolism-related genes between high and low macrophage abundance HCC patients (**c**) Venny plot of tumor-associated macrophage-related metabolic genes identified by Pearson correlation analysis

**Fig. 3:**
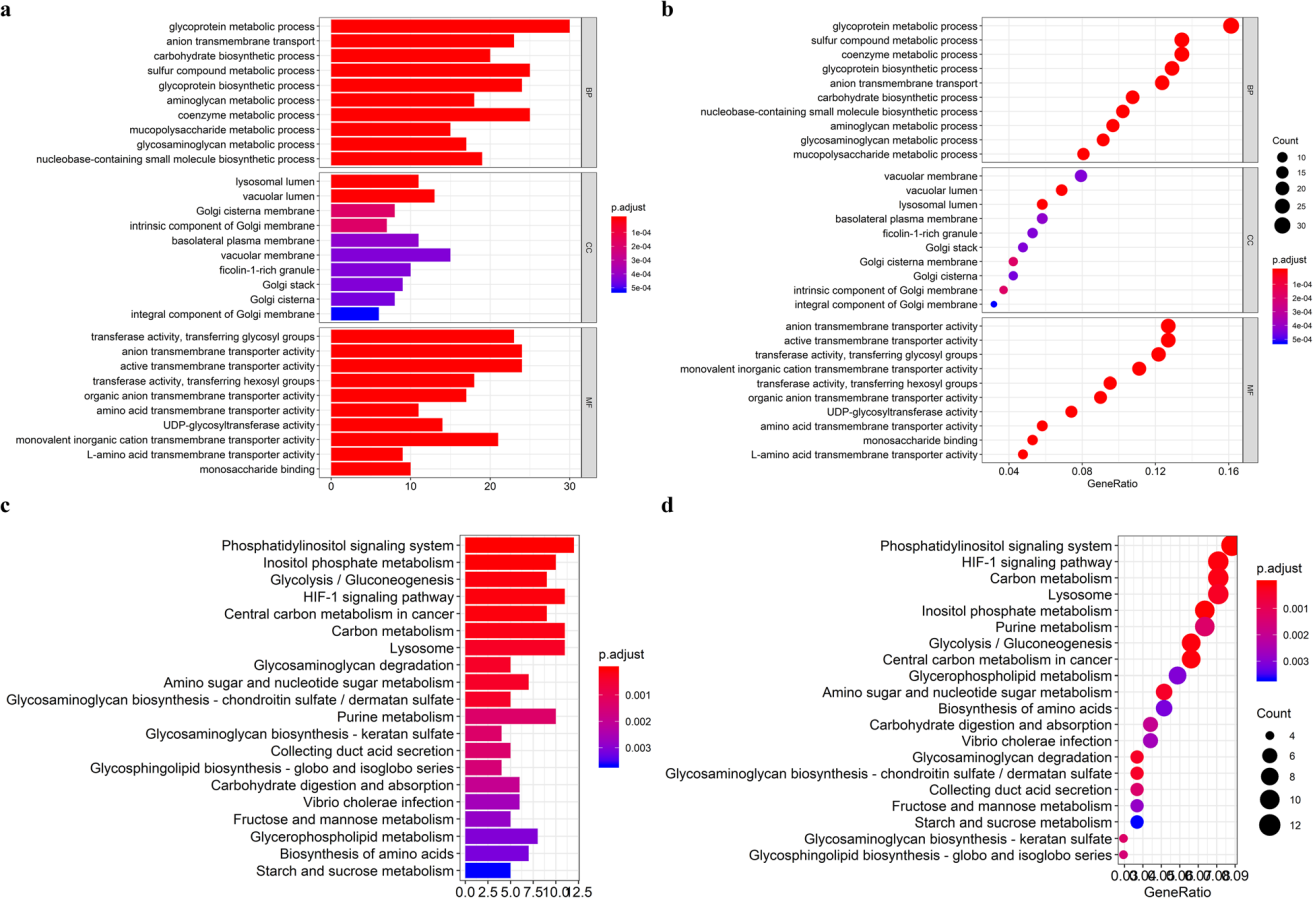
Functional enrichment analysis of tumor-associated macrophage-related metabolic genes (TRMGs). **a-b** GO enrichment analysis of TRMGs. **c-d** KEGG enrichment analysis of TRMGs

We observed that patients with a higher abundance of macrophage infiltration had a poorer prognosis (Fig. 2a), which prompted us to identify prognostic biomarkers of HCC according to the degree of macrophage infiltration. We identified 1382 metabolic genes with different expression levels between high macrophage infiltration and low macrophage infiltration patients (Fig. 2b), and 192 genes were significantly correlated with macrophage infiltration (cor > 0.4, *p* < 0.001) (Fig. 2c). KEGG and GO enrichment analysis demonstrates the involvement of these genes in many aspects of metabolism, including those related to glycoproteins, sulfur compounds, coenzymes, carbon, purines, glycolysis, and glycogenesis (Fig. 3).

### Construction of prognostic signature

**Fig. 4:**
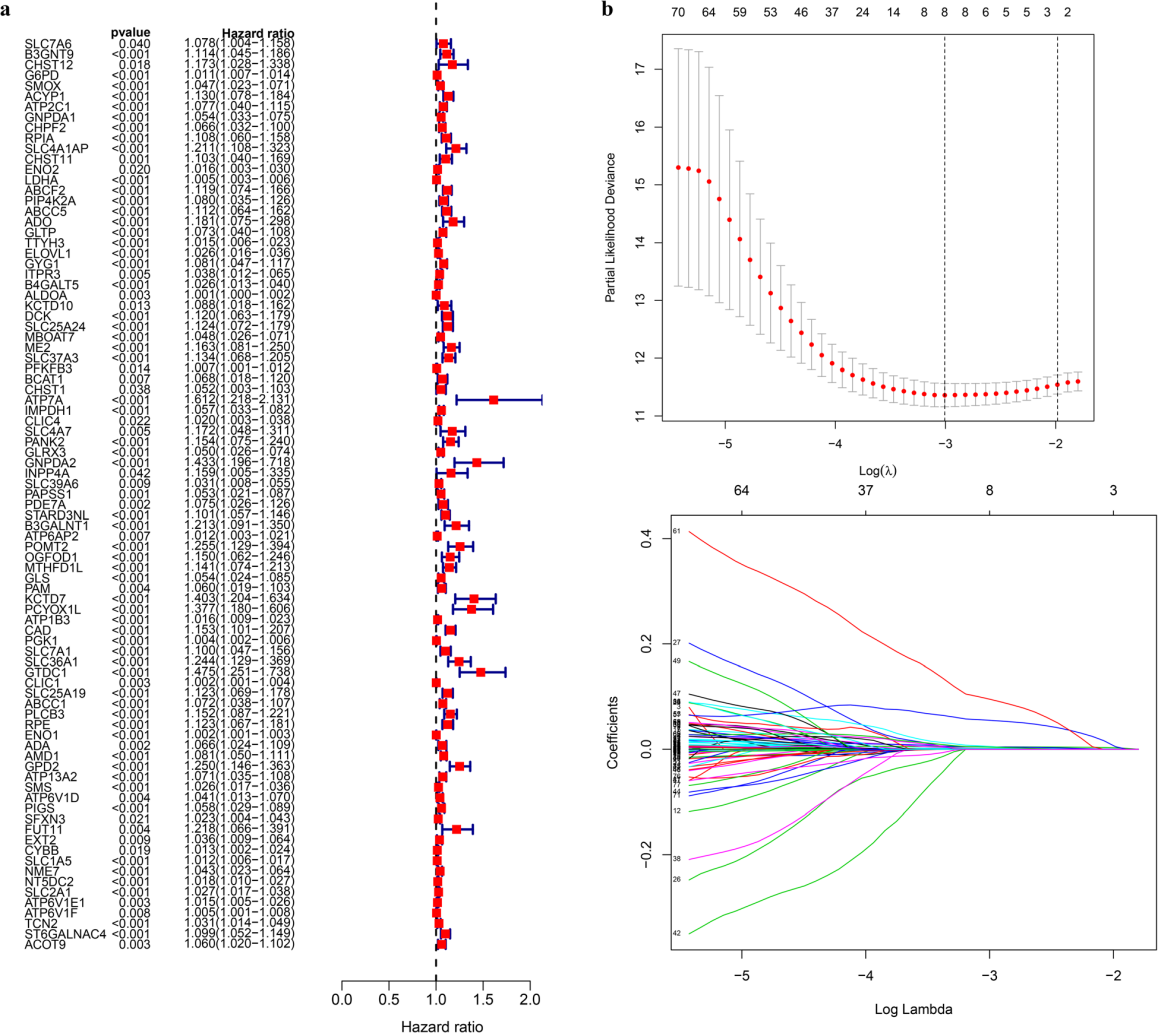
The building process of the prognostic signature. **a** Forrest plot of prognostic metabolic genes identified by Univariate Cox and Kaplan-Meier survival analysis. **b** Lasso regression analysis performed on prognostic metabolic genes

**Fig. 5:**
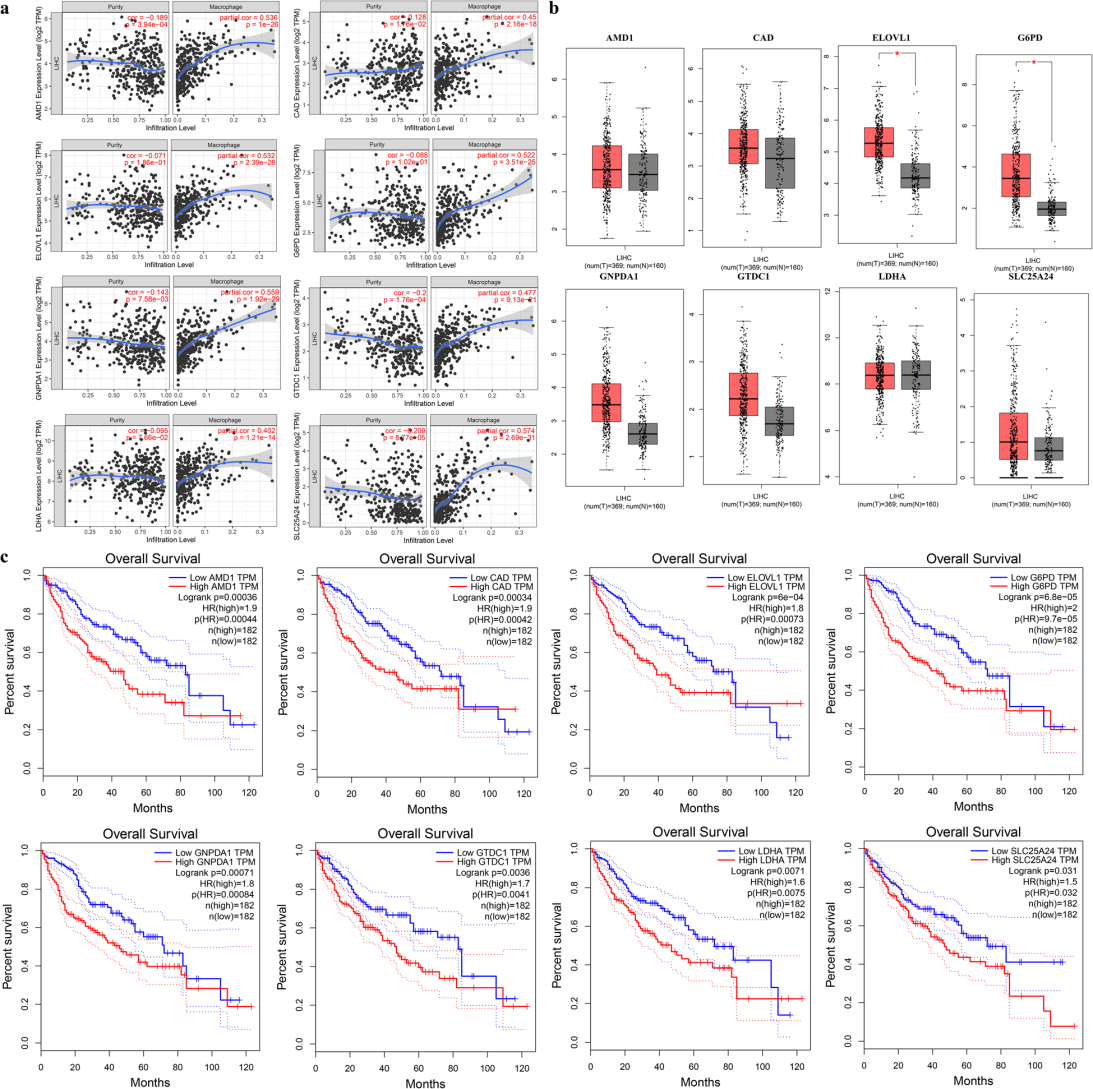
Eight genes that comprise the prognostic model. **a** Correlation analysis between expression level of 8 genes and macrophage infiltration level (image downloaded from https://cistrome.shinyapps.io/timer/). **b** Boxplot of expression level of 8 genes between tumor and normal tissues (image downloaded from http://gepia.cancer-pku.cn/). **c** Kaplan-Meier survival curve showing the relationship between the 8-gene signature and overall survival (image downloaded from http://gepia.cancer-pku.cn/)

A total of 87 TRMGs were identified by Kaplan-Meier survival analysis and Univariate Cox regression analysis, and all were risk factors for the overall survival of HCC (Fig. 4a). The lasso regression model included the following 8 metabolic genes associated with prognosis (Fig. 4b-c): G6PD, GNPDA1, LDHA, ELOVL1, SLC25A24, CAD, GTDC1, and AMD1. The risk score = 0.0045 * expression of G6PD + 0.0010 * expression of GNPDA1 + 0.0018 * expression of LDHA + 0.0042 * expression of ELOVL1 + 0.0025 * expression of SLC25A24 + 0.0519 * expression of CAD + 0.0847 * expression of GTDC1 + 0.0030 * expression of AMD1. With the median value of 0.731 as the critical value, two groups were obtained, low-risk and high-risk. The relationship between the expression of these 8 genes and macrophage cell infiltration is shown in Fig. 5.

### Prognostic assessment of the signature in the TCGA cohort

**Fig. 6:**
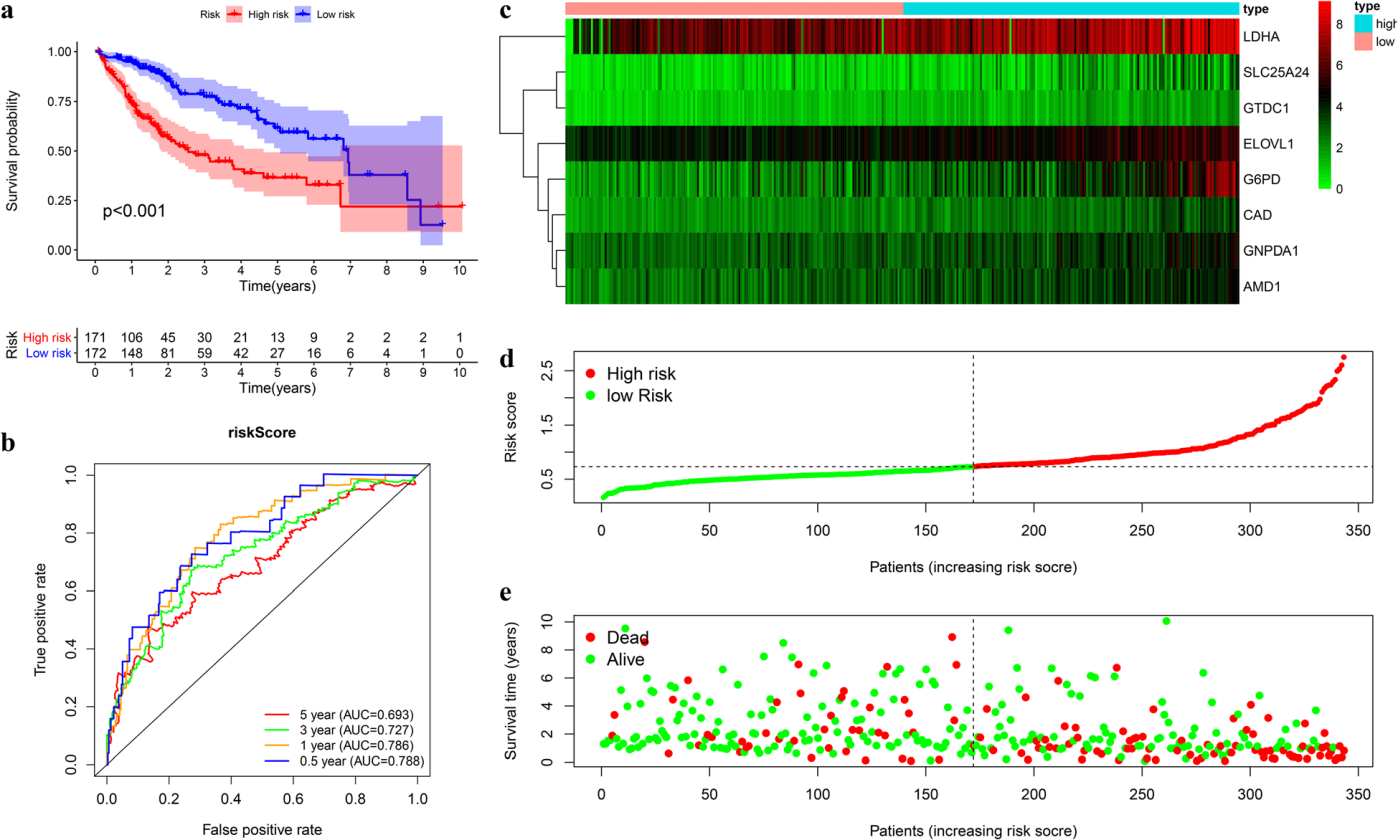
Construction of the prognostic model in TCGA cohort. **a-b** Kaplan-Meier survival analysis and time-dependent ROC analysis for predicting overall survival for patients in TCGA cohort used by risk score. **c-e** Heatmap of the eight genes and the distribution of risk score and the survival status of patients

**Fig. 7:**
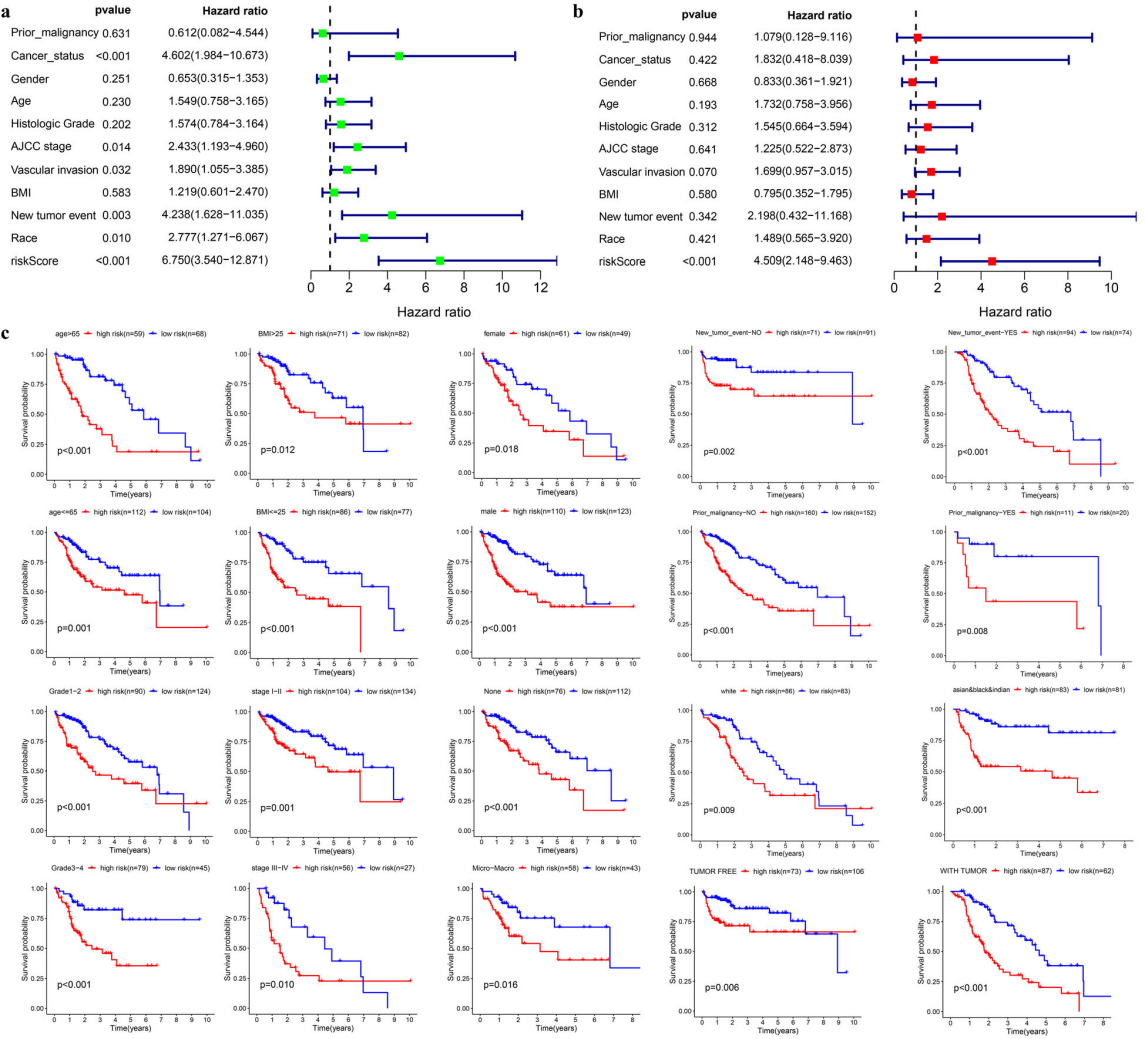
Internal validation of the prognostic model in TCGA cohort. **a-b** Univariate and multivariate regression analysis of the relationship between the RS and clinicopathological characteristics regarding overall survival in the TCGA cohort (green represents univariate analysis, and red represents multivariate analysis). **c** Subgroup survival analysis depending on different clinical features

Kaplan-Meier survival showed poorer overall survival (OS) of the high-risk group than the low-risk one (log-rank test *p* < 0.001) (Fig. 6a). The AUC for 1, 3, and 5-year OS was 0.786, 0.727, and 0.693, respectively (Fig. 6b). The survival status distribution map also showed that mortality increased as the risk score increased (Fig. 6c-e). Univariate and multivariate Cox regression analysis reveals the risk score as an independent prognosis predictor for OS (Fig. 7a-b).

### Internal validation of the prognostic signature in the TCGA cohort

Patients were assigned to 20 groups by clinical characteristics. The high-risk group exhibited a poorer prognostic outcome in each subgroup than the low-risk one based on the Kaplan-Meier analysis. All *p* values obtained from log-rank tests in each subgroup were less than 0.05 (Fig. 7c).

### External validation of the prognostic signature in ICGC cohort

**Fig. 8:**
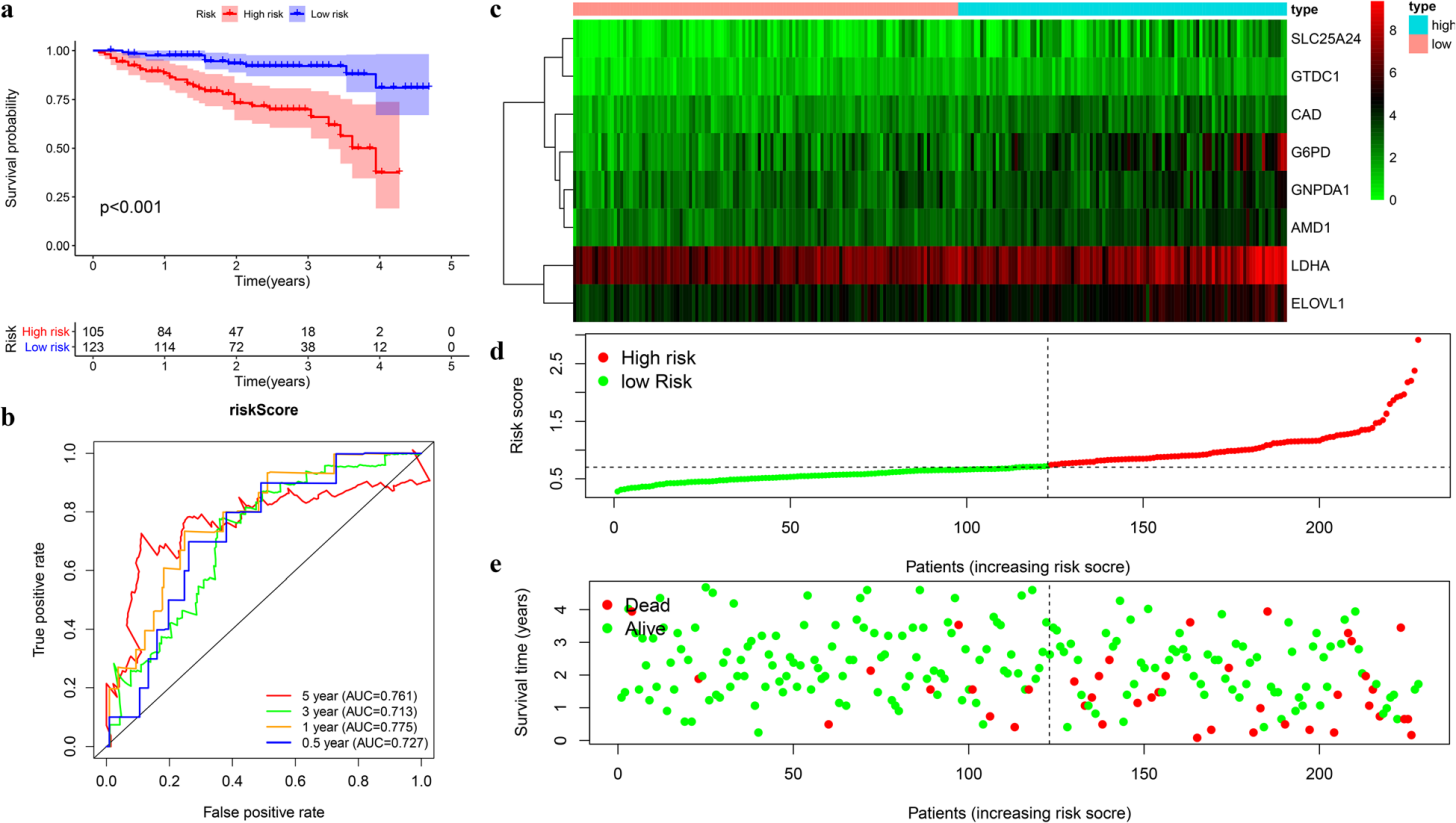
External validation of the prognostic model in ICGC cohort. **a-b** Kaplan-Meier survival analysis and time-dependent ROC analysis for predicting overall survival for patients validated in ICGC cohort using risk score (**c-e**). Heatmap of the eight genes and the distribution of risk score and the survival status of patients

**Fig. 9:**
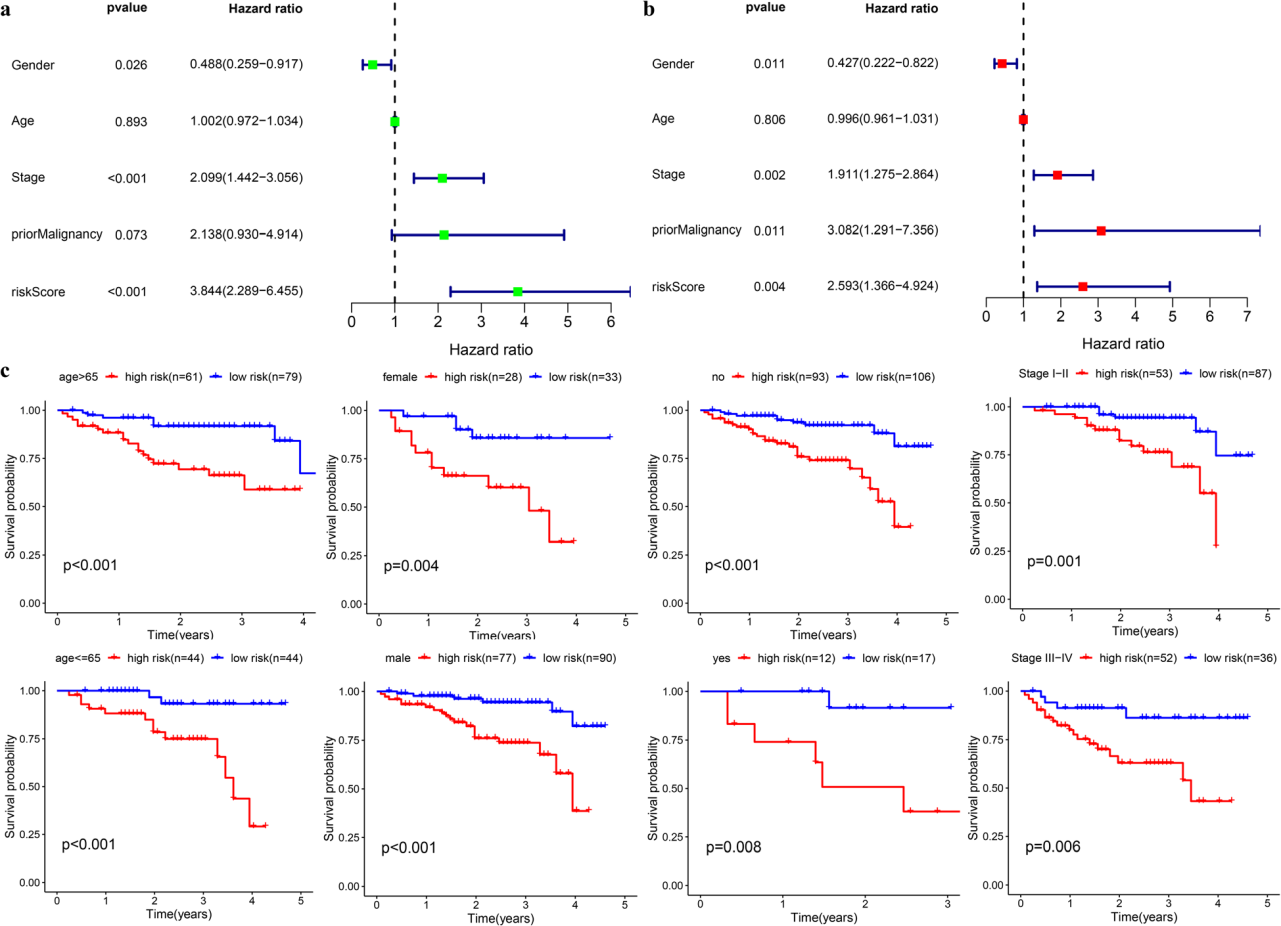
Verification of the universal applicability of the prognostic model in the ICGC cohort. **a-b** Univariate and multivariate regression analysis of the relationship between the RS and clinicopathological characteristics regarding overall survival in the ICGC cohort (green represents univariate analysis, and red represents multivariate analysis). **c** Subgroup survival analysis depending on different clinical features

A group of 228 patients with complete survival information served as an external validation cohort. The formula mentioned previously was used to calculate the risk score, and patients were assigned to two groups with the same cutoff (0.731). Similar to the TCGA cohort, the high-risk group showed worse survival as demonstrated by Kaplan-Meier survival analysis (*p* < 0.01) (Fig. 8a). The AUC for OS at 1, 3, and 5 years was 0.775, 0.713, and 0.761, respectively (Fig. 8b). Overall survival was independently predicted by the risk score as implied by Univariate and multivariate Cox regression analysis (Fig. 9a-b). Then patients assigned by clinical features (8 subgroups) were subject to Kaplan-Meier survival analysis, where the high-risk group exhibited worse OS in each subgroup (Fig. 9c). These results further confirmed the robustness of the signature in predicting overall survival of HCC.

### Relationship between risk score and clinical features

According to the results of chi-square test, the high-risk group has higher histopathological grade, later clinical stage, greater vascular invasion, and higher probability of new tumor growth after initial treatment, and this group usually presents with a tumor, which may help to explain the reasons leading to the poor prognosis of high-risk group (Tables 1-2).

**Table 1:** The chi-square test of the relationship between risk score and clinical features in TCGA cohort

Clinical feature	Risk Score		c2	P
	High risk n(%)	Low risk n(%)		
Age			0.931	0.335
>65	59 (46.46%)	68 (53.54%)		
≤ 65	112 (51.85%)	104 (48.15%)		
BMI			1.275	0.259
>25	71 (46.41%)	82 (53.59%)		
≤ 25	86 (52.76%)	77 (47.24%)		
Family cancer history			0.149	0.7
NO	29 (23.58%)	94 (76.42%)		
YES	19 (26.03%)	54 (73.97%)		
Gender			2.032	0.154
female	61 (55.45%)	49 (44.55%)		
male	110 (47.21%)	123 (52.79%)		
Tumor status			10.08931	0.001
tumor free	73 (40.78%)	106 (59.22%)		
with tumor	87 (58.39%)	62 (41.61%)		
New tumor event			4.85	0.028
no	71 (43.83%)	91 (56.17%)		
yes	94 (55.95%)	74 (44.05%)		
Prior Malignancy			2.815	0.093
no	160 (51.28%)	152 (48.72%)		
yes	11 (35.48%)	20 (64.52%)		
Histologic Grade			14.724	<0.001
G1–2	90 (42.06%)	124 (57.94%)		
G3–4	79 (63.71%)	45 (36.29%)		
Stage			13.911	<0.001
I-II	104 (43.70%)	134 (56.30%)		
III-IV	56 (67.47%)	27 (32.53%)		
Vascular tumor			9.806	0.002
none	76 (38.38%)	122 (61.62%)		
Micro-Macro	58 (57.43%)	43 (42.57%)		
Race			0.003	0.96
white	86 (50.89%)	83 (49.11%)		
asian-blank&Indian	83 (50.61%)	81 (49.39%)

**Table 2:** The chi-square test of the relationship between risk score and clinical features in ICGC cohort

Clinical feature	Risk Score		c^2^	P
	High risk n(%)	Low risk n(%)		
Age			0.899	0.343
>65	61 (43.57%)	79 (56.43%)		
≤ 65	44 (50%)	44 (50%)		
Gender			0.001	0.978
female	28 (45.91%)	33 (54.10%)		
male	77 (46.11%)	90 (53.89%)		
Prior Malignancy			0.292	0.589
no	93 (46.73%)	106 (53.27%)		
yes	12 (41.38%)	17 (58.62%)		
Stage			9.806	0.002
I-II	53 (37.32%)	87 (62.68%)		
III-IV	52 (59.09%)	36 (40.91%)		

### Relationship between risk score and immune infiltration

**Fig. 10:**
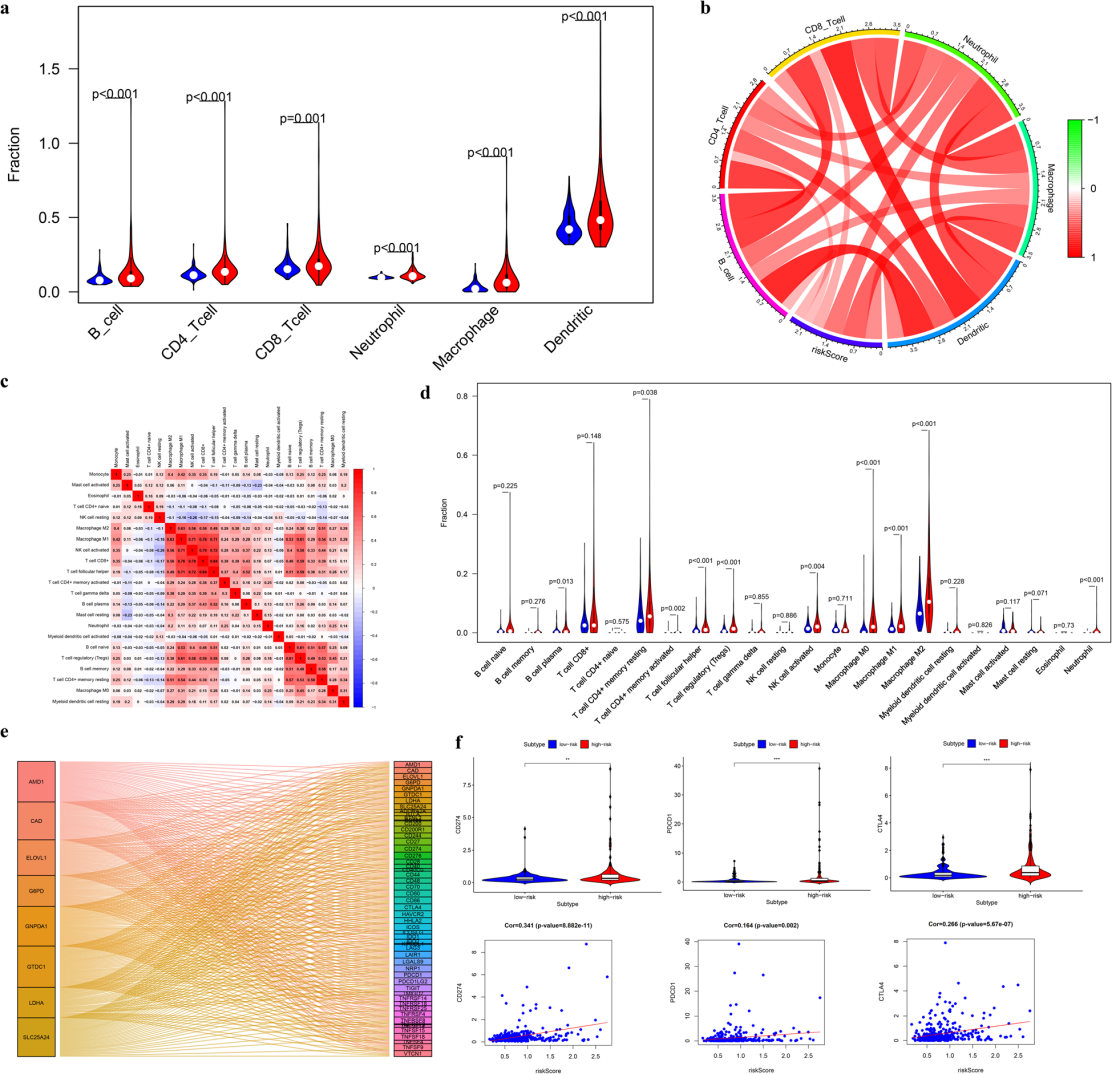
The landscape of immune infiltration in high- and low-risk HCC patients. **a-b** Relationships between the risk score and infiltration abundance of six types of immune cells (TIMER method, red represents high-risk group, blue represents low-risk group). **c** Cor-heatmap of absolute abundance of 22 immune infiltration cell proportions estimated using CIBERSORT-ABS method. **d** Vioplot of absolute abundance of 22 immune infiltration cell estimated by CIBERSORT-ABS method (red represents high-risk group, blue represents low-risk group). **e** Analysis of coexpression of 8 genes and immune checkpoints (**f**) The comparison of expression levels of CD274 (PDL1), PDCD1, and CTLA4 between different risk groups and correlation analysis between risk score and the expression levels of CD274 (PDL1), PDCD1, and CTLA4

The TIMER algorithm showed that the high-risk group presented greater infiltration of 6 types of immune cells, relative to the low-risk group (Fig. 10a). Among these immune cells, the risk score has the strongest correlation with macrophages and neutrophils (Fig. 10b). Macrophages and neutrophils were also estimated to have greater infiltration in the high-risk group based on the CIBERSORT-ABS algorithm (Fig. 10c-d). We also found that a variety of immune checkpoints correlated with the expression of 8 genes and that the expression of CD274, PDCD1, and CTLA4 (Fig. 10e-f) was positively correlated with the risk score.

### GSEA between different risk groups

Five representative upregulated signaling pathways in the groups with low and high risk, respectively, were identified by the NES score from TCGA and ICGC cohorts (Fig. S1). Interestingly, the high-risk group presents significantly lower metabolic activity than the low-risk group in the high-risk group in these two independent cohorts (Fig. S1, Table S1–2).

## Discussion

Hepatocellular carcinoma (HCC) is a representative type of primary liver cancer. Despite recent improvements in treatment, it still holds a low five-year survival [20]. The mechanisms underlying the initiation and development of HCC remain to be clarified. In recent years, the initiation and development of HCC has been shown to be not only related to the characteristics of the tumor itself but also to its microenvironment [3]. Tumor and stromal cells, various immune inflammatory cells, chemokines, and cytokines together constitute the tumor microenvironment [21], and tumor-associated macrophages (TAMs) play important roles as significant inflammatory cells. As is increasingly shown by several studies, TAMs promote the generation, metastasis, and immunosuppression of HCC [22, 23]. However, how TAMs promote tumorigenesis, growth, invasion, and metastasis, how TAMs can lead to immunosuppression, and how tumor cells interact with TAMs remain unresolved topics. With the development of genomics and proteomics, we can better study the molecular mechanisms by which TAMs influence tumorigenesis and development, clarify the relationship between TAMs and tumors, and provide new clues for tumor biotherapy. It has been reported that the abnormal metabolism of malignant tumor cells can not only induce changes in the phenotype and function of TAMs but can also change the metabolic mode of TAMs, causing them to exert immunosuppressive activity and to ultimately promote the development and metastasis of tumors [12]. On that account, exploring the metabolic changes of tumor cells and TAMs and their intricate relationship is necessary.

This study found that the HCC patients with higher levels of macrophage infiltration had poorer prognosis. We identified metabolism-related genes with expression levels closely correlated with macrophage infiltration. A prognostic signature containing 8 genes was established using lasso regression analysis, and this signature correlated with the overall survival of HCC patients in TCGA and ICGC cohorts. The signature was applicable for people with different clinical features, demonstrating that this signature is robust. Additionally, the signature could be used as an independent predictor for overall survival of HCC as confirmed by Cox analysis. The immune microenvironment of different risk groups was also evaluated; immune infiltration has previously been reported to be correlated with clinical outcome for many kinds of cancer [24]. In this study, we found that the high-risk patients with unfavorable prognosis had higher levels of macrophage infiltration, which is consistent with the results of Li [25]. In addition, compared with the low-risk group, the high-risk one exhibited a higher infiltration level of Tregs and neutrophils; previous literature also demonstrated the negative correlation of prognosis in HCC with the infiltration of these two immune cell types [26, 27]. However, we also found that the infiltration level of B cells, CD4 T cells, and CD8 T cells was higher in the high-risk group than in the low-risk group using the TIMER database, which contradicts previous studies [28–30]. The two groups showed no difference in the infiltration of these three immune cell types as found in the CIBERSORT-ABS method. PDL1 (CD274), PDCD1, and CTLA4 are three immune check-point markers commonly analyzed in the clinic [31], and these proteins may be used by tumors to escape immune surveillance by controlling cell cycle progression and extracellular and intracellular signals. The positive correlation of the risk score with the expression levels of PDL1 (CD274), PDCD1, and CTLA4 was found, suggesting that metabolic reprogramming genes may have a significant effect on the tumor immune microenvironment. Interestingly, we found that the high-risk group had significantly lower activity in metabolic pathways than the low-risk group in two independent cohorts based on GSEA. This finding is consistent with the improved prognosis for the metabolism subgroup of HCC reported by Gao et al. [15].

Lactate dehydrogenase A (LDHA) is a metabolic enzyme that can produce lactate in human body, and it has become an important indicator of clinical tumor diagnosis. It has been reported that LDHA can mediate tumor immune escape by inhibiting the activity of T cells and NK cells [32]. LDHA expression levels are positively correlated with macrophage abundance in HCC, which also provides a clue for the further study of the tumor immune mechanisms regulated by LDHA. The human solute carrier protein 25 family is a superfamily of human solute carrier proteins, which play a role in molecular transport, oxidative phosphorylation, and iron metabolism related to urea and the citric acid cycle. Recently, several studies have shown that SLC25 family members can affect tumor initiation and development [33]. This study demonstrates for the first time the correlation among SLC25A24, HCC prognosis, and macrophage infiltration. Glycosyltransferase is crucial in glycosylation; it catalyzes the transfer of an active glycosyl group from a glycosyl donor to a glycosyl receptor and forms glycosidic bonds [34]. David Kessel et al. found that the levels of three plasma glycosyltransferases could affect cancer patients’ neoplasia, especially for patients with tumors metastasizing to the liver [35]. However, the mechanism of glycosyltransferase-like domain containing 1 (GTDC1) in HCC remains to be elucidated. After HCC leads to the unlimited proliferation of hepatocytes, the speed of development begins to slow. The most common metabolic phenomenon observed in HCC cells is an increased glycolysis rate, which is known as the Warburg effect [36]. Glucose-6-phosphate dehydrogenase (G6PD) is an important metabolic enzyme in glycolysis, and it is correlated with the proliferation and apoptosis of HCC [37]. This study is the first to identify correlation between 6-phosphate dehydrogenase and the abundance of tumor macrophages, which will provide a new direction for further exploration of the mechanism of G6PD in regulating HCC development. The rapid growth of tumors also depends on the polyamine content in cells. The abnormal metabolism of polyamine can also cause malignant transformation of cells [38]. The metabolism of polyamine has attracted special attention in the tumor research field. Known as SAMDC, S-adenosylmethionine decarboxylase 1 (AMD1) is the rate limiting enzyme, which regulates polyamine metabolism [39, 40]. In lymphoma, AMD1 acts as a tumor suppressor gene by regulating the posttranslational modification of eukaryotic translation initiation factor 5A (eIF5A) [39]. In prostate cancer, AMD1 regulates the mTOR pathway to influence tumor cell proliferation, thus promoting tumor development [41]. AMD1 also remarkably affects breast cancer initiation and development [42]. However, the expression and role of AMD1 in HCC are not commonly reported. The role of the remaining three genes, CAD, GNPDA1, and ELOVL1, in tumors remains unclear.

Our study is the first attempt to identify the metabolic genes with prognostic significance for HCC from the perspective of immune infiltration. Our results show a significant relationship between metabolic reprogramming and the tumor immune microenvironment, and metabolic disorders may affect tumor development by mediating tumor immune regulation. These results provide theoretical support for exploring the nonmetabolic mechanisms of metabolic genes in the future. However, there are some limitations in our research. We have not performed further experiments to explore the immune mechanisms of these metabolic genes, but we will address this in the future work. And as for the HCC patients who are mostly diagnosed by imaging modalities and treated with nonsurgical methods, the value of this model may be limited, because our model needs to quantify the expression levels of eight specific genes in tumor tissues. Our research preliminarily demonstrates the feasibility of exploring the immune activity of tumors from the perspective of metabolic reprogramming. Therefore, it is necessary to continue performing multicenter prospective research on this subject.

## Conclusion

To evaluate the prognosis of HCC, our study established a novel risk score by examining how tumor macrophages correlated with metabolic genes. Considering the heterogeneity of HCC, the prognostic evaluation of HCC may be improved by the prognostic model. Our study also provides theoretical support for further elucidating the complex relationship between metabolic reprogramming and tumor immune mechanisms.

## Supplementary Information

**Additional file 1: Table S1.** Five representative upregulated pathways in high-risk and low-risk groups from TCGA datasets.

**Additional file 2: Table S2.** Five representative upregulated pathways in high-risk and low-risk groups from ICGC datasets.

**Additional file 3:** **Figure S1.** GSEA between different risk groups. (a) Five representative upregulated pathways in the high-risk groups from TCGA datasets. (b) Five representative upregulated pathways in the low-risk groups from TCGA datasets. (c) Five representative upregulated pathways in the high-risk groups from the ICGC datasets. (d) Five representative upregulated pathways in the low-risk groups from the ICGC datasets.

## Data Availability

The datasets analysed for this study were obtained from The Cancer Genome Atlas (TCGA)(https://portal.gdc.cancer.gov/) and International Cancer Genome Consortium (ICGC) (https://icgc.org/) and TIMER (https://cistrome.shinyapps.io/timer/) and UCSC Xena website (https://xenabrowser.net/).

## Abbreviations

HCC: Hepatocellular carcinoma
TCGA: The Cancer Genome Atlas
ICGC: International Cancer Genome Consortium
TAMs: Tumor associated macrophages
TRMGs: TAM related metabolic genes
DEGs: Differentially expressed genes
GO: Gene Ontology
KEGG: Kyoto Encyclopedia of Genes and Genomes
ROC: Receiver operating characteristic
OS: Overall survival
LASSO: Least absolute shrinkage and selection operator
FDR: False discovery rate
AUC: Area under curve
GSEA: Gene Set Enrichment Analysis
NES: Normalized enrichment score
