# Supplementary material for: Eight-gene metabolic signature related with tumor-associated macrophages predicting overall survival for hepatocellular carcinoma
Source: BMC Cancer. 2021 Jan 7;21:31. doi: 10.1186/s12885-020-07734-z (PMC7789516; doi:10.1186/s12885-020-07734-z)
Supplement: Supplementary file 1 — Additional file 1: Table S1. Five representative upregulated pathways in high-risk and low-risk groups from TCGA datasets. [file 12885_2020_7734_MOESM1_ESM.docx]

Table S1 The five representative pathways up-regulated in high- and low-risk groups of TCGA datasets

|  | TCGA |  |  |
| --- | --- | --- | --- |
| High_risk | NES | NOM p-val | FDR q-val |
| KEGG_OOCYTE_MEIOSIS | 2.2584715 | 0 | 0 |
| KEGG_UBIQUITIN_MEDIATED_PROTEOLYSIS | 2.1949232 | 0 | 0 |
| KEGG_PROGESTERONE_MEDIATED_OOCYTE_MATURATION | 2.1891253 | 0 | 0 |
| KEGG_SMALL_CELL_LUNG_CANCER | 2.1504967 | 0 | 0 |
| KEGG_NEUROTROPHIN_SIGNALING_PATHWAY | 2.1110988 | 0 | 0 |
| Low_risk |  |  |  |
| KEGG_DRUG_METABOLISM_CYTOCHROME_P450 | -2.0892992 | 0 | 5.41E-04 |
| KEGG_PRIMARY_BILE_ACID_BIOSYNTHESIS | -2.0672688 | 0 | 8.03E-04 |
| KEGG_RETINOL_METABOLISM | -2.016827 | 0 | 0.001733808 |
| KEGG_FATTY_ACID_METABOLISM | -1.9784331 | 0 | 0.002183813 |
| KEGG_GLYCINE_SERINE_AND_THREONINE_METABOLISM | -1.9253879 | 0.001937985 | 0.003704905 |
