# Supplementary material for: Eight-gene metabolic signature related with tumor-associated macrophages predicting overall survival for hepatocellular carcinoma
Source: BMC Cancer. 2021 Jan 7;21:31. doi: 10.1186/s12885-020-07734-z (PMC7789516; doi:10.1186/s12885-020-07734-z)
Supplement: Supplementary file 2 — Additional file 2: Table S2. Five representative upregulated pathways in high-risk and low-risk groups from ICGC datasets. [file 12885_2020_7734_MOESM2_ESM.docx]

Table S2 The five representative pathways up-regulated in high- and low-risk groups of ICGC datasets

|  | ICGC |  |  |
| --- | --- | --- | --- |
| High_risk | NES | NOM p-val | FDR q-val |
| KEGG_VASOPRESSIN_REGULATED_WATER_REABSORPTION | 1.932376 | 0.001937985 | 0.21499497 |
| KEGG_SPLICEOSOME | 1.927817 | 0 | 0.11686785 |
| KEGG_RNA_DEGRADATION | 1.882863 | 0 | 0.12899259 |
| KEGG_HOMOLOGOUS_RECOMBINATION | 1.874749 | 0 | 0.10589986 |
| KEGG_UBIQUITIN_MEDIATED_PROTEOLYSIS | 1.860979 | 0.001956947 | 0.10060089 |
| Low_risk |  |  |  |
| KEGG_RETINOL_METABOLISM | -2.22568 | 0 | 3.55E-04 |
| KEGG_DRUG_METABOLISM_CYTOCHROME_P450 | -2.13035 | 0 | 6.70E-04 |
| KEGG_FATTY_ACID_METABOLISM | -2.10359 | 0 | 9.18E-04 |
| KEGG_METABOLISM_OF_XENOBIOTICS_BY_CYTOCHROME_P450 | -2.01529 | 0.001865672 | 0.004965445 |
| KEGG_PPAR_SIGNALING_PATHWAY | -2.00926 | 0.001904762 | 0.004488035 |
