# Supplementary figures and images for: Eight-gene metabolic signature related with tumor-associated macrophages predicting overall survival for hepatocellular carcinoma
Source: BMC Cancer. 2021 Jan 7;21:31. doi: 10.1186/s12885-020-07734-z (PMC7789516; doi:10.1186/s12885-020-07734-z)

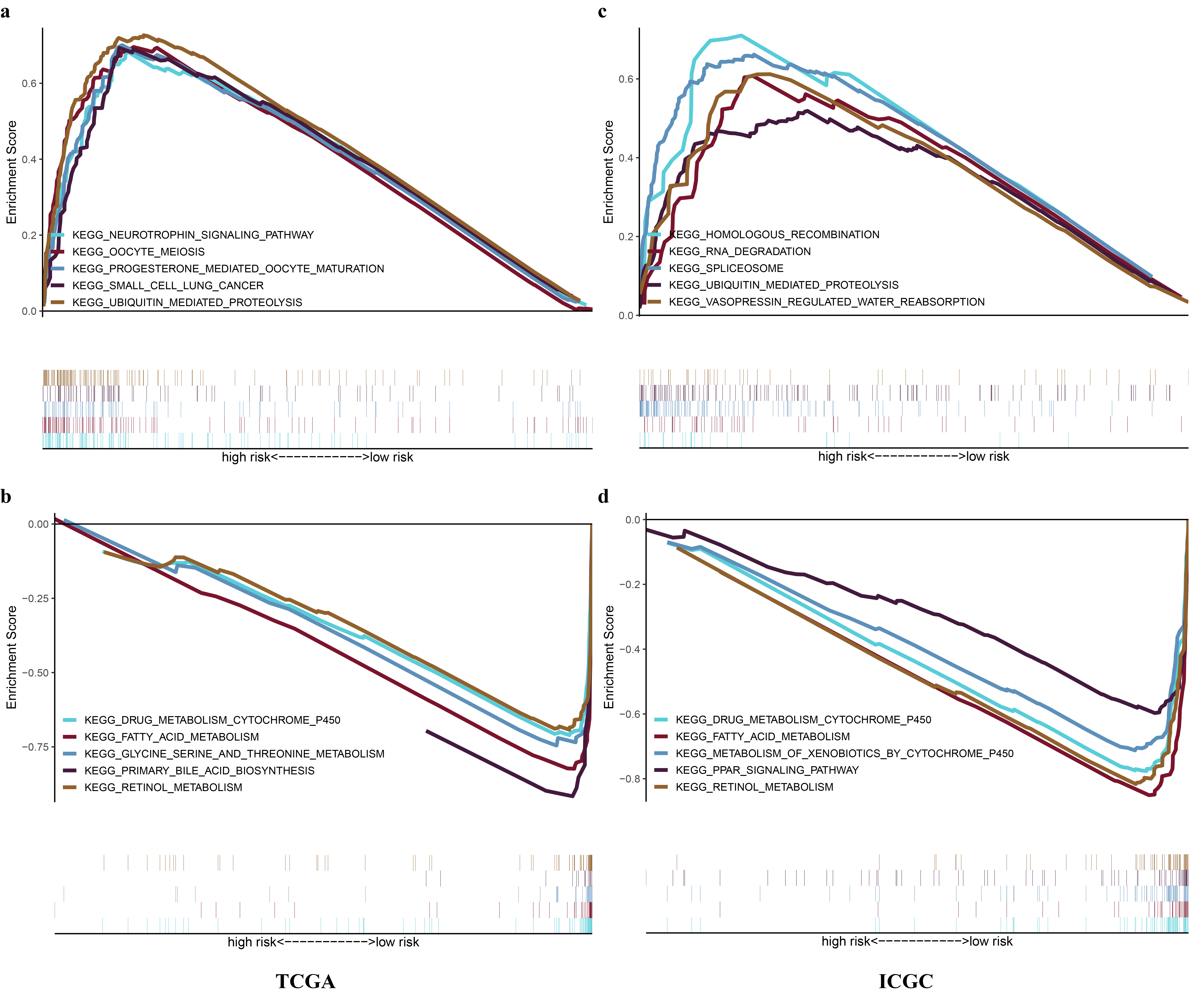

Supplement: Supplementary file 3 — Additional file 3: Figure S1. GSEA between different risk groups. (a) Five representative upregulated pathways in the high-risk groups from TCGA datasets. (b) Five representative upregulated pathways in the low-risk groups from TCGA datasets. (c) Five representative upregulated pathways in the high-risk groups from the ICGC datasets. (d) Five representative upregulated pathways in the low-risk groups from the ICGC datasets. [file 12885_2020_7734_MOESM3_ESM.tif]
